# Electrochemically Triggered Energy Release from an Azothiophene‐Based Molecular Solar Thermal System

**DOI:** 10.1002/cssc.202200958

**Published:** 2022-07-27

**Authors:** Evanie Franz, Anne Kunz, Nils Oberhof, Andreas H. Heindl, Manon Bertram, Lukas Fusek, Nicola Taccardi, Peter Wasserscheid, Andreas Dreuw, Hermann A. Wegner, Olaf Brummel, Jörg Libuda

**Affiliations:** ^1^ Interface Research and Catalysis Erlangen Center for Interface Research and Catalysis Friedrich-Alexander-Universität Erlangen-Nürnberg Egerlandstraße 3 91058 Erlangen Germany; ^2^ Institute of Organic Chemistry Justus-Liebig-Universität Heinrich-Buff-Ring 17 35392 Giessen Germany; ^3^ Interdisciplinary Center for Scientific Computing Universität Heidelberg Im Neuenheimer Feld 205 A 69120 Heidelberg Germany; ^4^ Institute of Chemical Reaction Engineering Friedrich-Alexander-Universität Erlangen-Nürnberg Egerlandstraße 3 D-91058 Erlangen Germany; ^5^ Forschungszentrum Jülich GmbH Helmholtz Institute Erlangen-Nürnberg for Renewable Energy Egerlandstraße 3 D-91058 Erlangen Germany

**Keywords:** Electrochemistry, Energy Storage, Photochemistry, Photoswitches, Solar Thermal Fuels

## Abstract

Molecular solar thermal (MOST) systems combine solar energy conversion, storage, and release in simple one‐photon one‐molecule processes. Here, we address the electrochemically triggered energy release from an azothiophene‐based MOST system by photoelectrochemical infrared reflection absorption spectroscopy (PEC‐IRRAS) and density functional theory (DFT). Specifically, the electrochemically triggered back‐reaction from the energy rich (*Z*)‐3‐cyanophenylazothiophene to its energy lean (*E*)‐isomer using highly oriented pyrolytic graphite (HOPG) as the working electrode was studied. Theory predicts that two reaction channels are accessible, an oxidative one (hole‐catalyzed) and a reductive one (electron‐catalyzed). Experimentally it was found that the photo‐isomer decomposes during hole‐catalyzed energy release. Electrochemically triggered back‐conversion was possible, however, through the electron‐catalyzed reaction channel. The reaction rate could be tuned by the electrode potential within two orders of magnitude. It was shown that the MOST system withstands 100 conversion cycles without detectable decomposition of the photoswitch. After 100 cycles, the photochemical conversion was still quantitative and the electrochemically triggered back‐reaction reached 94 % of the original conversion level.

## Graphical abstract

**Molecular Solar Thermal (MOST)**: MOST systems combine solar energy conversion, storage, and release in a single molecule. One class of MOST systems, azothiophenes, could be photo‐isomerized from the (*E*) into the high‐energy (*Z*)‐state. Triggering the energy release electrochemically enabled a direct control of the release rate and provided excellent cyclability.

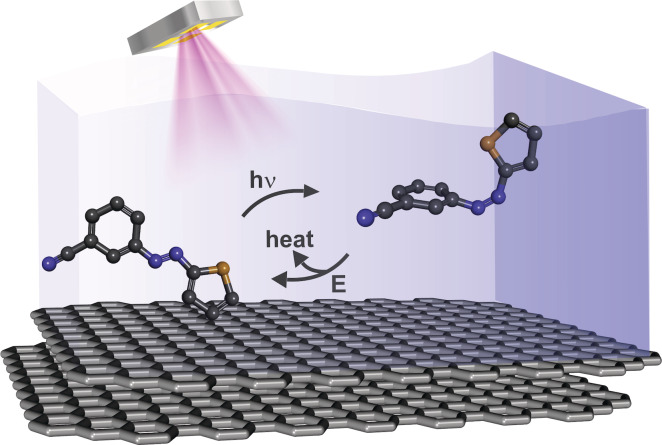

## Introduction

Molecular Solar Thermal (MOST) systems enable us to combine solar energy conversion, storage, and release in simple one‐photon one‐molecule processes.[^1^, ^2^, ^3^, ^4^, ^5^, ^6^] MOST systems are based on photoactive compounds (photoswitches) in which photo‐isomerization towards a high‐energy species occurs upon the absorbance of solar energy.[^7^, ^8^, ^9^] Prominent examples of MOST systems are the quadricyclane/norbornadiene (NBD/QC) couple,[^10^, ^11^, ^12^, ^13^, ^14^, ^15^] the dihydroazulene/vinylheptafulvene (DHA/VHF) couple,^[16]^ the (*E*)/(*Z*)‐azobenzene (*E*/*Z*‐AZO) couple[^4^, ^6^, ^7^, ^8^, ^17^, ^18^, ^19^, ^20^] and their derivatives. In the field of *E*/*Z*‐AZOs, heteroarylazobenzenes (HetABs) in general and azothiophenes in specific, have attracted particular interest as their physicochemical properties (i. e. absorption range^[21]^ and thermal half‐life[^21^, ^22^, ^23^]) often outperform conventional *E*/*Z*‐AZOs.[^21^, ^24^] Azothiophenes benefit from a stabilisation of the (*Z*)‐state due to lone pair⋅⋅⋅π interactions caused by the sulfur atom facing to the aryl ring in an orthogonal geometry. The compounds show impressive fatigue resistance and photo‐isomerization efficiency. In addition, the absorption bands of the (*E*)‐ and (*Z*)‐isomers are favourably separated allowing for highly selective photo‐conversion.^[25]^

For the implementation of MOST systems into operational devices, it is essential to control the energy release. Several strategies exist to trigger the energy release, for example through thermal,[^5^, ^8^] optical,[^6^, ^8^, ^19^] or catalytical^[20]^ pathways. A particularly intriguing concept is to trigger the energy release of MOST systems electrochemically.[^11^, ^12^, ^13^, ^17^, ^26^, ^27^, ^28^, ^29^] While the isomerization reaction itself is not a redox‐reaction, charged intermediates may still initiate a chain reaction which drives the isomerisation.[^17^, ^26^, ^27^, ^29^] In previous work, some of the authors demonstrated for NBD‐based MOST systems that this approach enables direct control of the reaction kinetics.^[26]^ In stability tests over 1000 storage cycles, a reversibility of up to 99.8 % was achieved.^[11]^ For the *E*/*Z*‐AZO system, Hecht and co‐workers showed that the energy release can be triggered using a reductive (electron‐catalyzed),^[17]^ and an oxidative (hole‐catalyzed) reaction channel.^[27]^ While the reductive channel is accessible electrochemically, the oxidative reaction channel leads to irreversibly oxidized products when initiated electrochemically.^[27]^ Recently, Greenfield et al. managed to overcome this limitation by molecular design using azoheteroarene derivatives.^[29]^

In this work, we address the electrochemically triggered energy release from azothiophenes by photoelectrochemical infrared reflection absorption spectroscopy (PEC‐IRRAS) and density functional theory (DFT). The development of PEC‐IRRAS^[13]^ enables us to monitor the storage and release in situ over a large number of cycles.[^11^, ^12^] Spectroscopically, we identify the different isomers by the chemical fingerprint of their IR spectrum.[^11^, ^12^, ^13^, ^26^] In this work, we study the derivative 3‐cyanophenylazothiophene, which we identified to be one of the most promising candidates for MOST applications based on a systematic screening of different azothiophene derivatives.^[19]^ While the photoswitch decomposes in the oxidative reaction channel, we show that the reductive channel is efficient and selective. In reductive electrochemical triggering, the azothiophene withstands 100 conversion cycles without detectable decomposition of the photoswitch. To the best of our knowledge, this is the first study, which addresses the electrochemically triggered energy release of an azothiophene‐based MOST system.

## Results and Discussion

**Figure 1:**
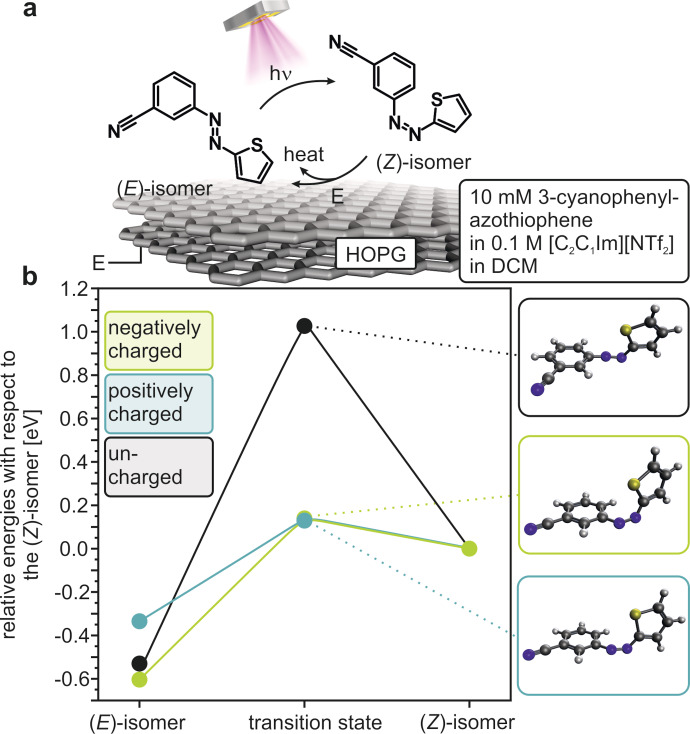
a) Schematic sketch of the investigated system: (*E*)/(Z)‐3‐cyanophenylazothiophene in DCM on HOPG. b) Calculated relative energies with respect to the (*Z*)‐isomer of the isomers and transition states.

We studied the photochemical conversion and the electrochemical back‐conversion of 3‐cyanophenylazothiophene by PEC‐IRRAS using the experimental conditions illustrated schematically in Figure 1a. We performed PEC‐IRRAS measurements with a solution of 10 mm (*E*)‐3‐cyanophenylazothiophene dissolved in dichloromethane (DCM). As the UV light source, we used a high‐power LED (2350 mW) with a wavelength of *λ*_max_=365 nm, which perfectly matches the absorption maximum of (*Z*)‐cyanophenylazothiophene (362 nm).^[19]^ We used highly oriented pyrolytic graphite (HOPG) as working electrode and 0.1 m [C_2_C_1_Im][NTf_2_] as supporting electrolyte. Note that the 3‐cyanophenylazothiophene system reacts under the experimental conditions with some of the most common supporting electrolytes for organic solvents (Bu_4_NClO_4_, Bu_4_NPF_6_, Bu_4_NBF_4_; see Supporting Information for details and a mechanistic discussion). We choose HOPG as chemically inert working electrode material. For NBD based MOST systems we demonstrated that metallic electrode materials, such as Pt, are catalytically active.^[30]^ This activity may, however, also lead to undesired side reactions, limiting the reversibility of the system.^[28]^

Before the in situ measurements, we first calculated the energetic profiles of the oxidative and reductive reaction channels by DFT. Specifically, we determined the total and relative energies of the (*E*)‐ and (*Z*)‐isomer, as well as the transition state. We calculated these values for the neutral, the positively and negatively charged species (Table S1 in the Supporting Information) for the untwisted conformers. Note that both isomers can adopt an “untwisted” and a “twisted” conformation. Thereby, “twisted” and “untwisted” are the conformers with the sulfur atom of the thiophene in trans and cis position, respectively. DFT calculations and comparisons of calculated and experimental IR spectra demonstrate, however, that the untwisted conformers are dominant (≥99.5 %) for both the (*E*) and the (*Z*)‐isomer under our experimental conditions (see Section 2 in Supporting Information for details). In Figure 1b we plotted the corresponding relative energies with respect to the corresponding (*Z*)‐isomer as a function of the oxidation state.

We observe for all charge states that the (*E*)‐isomer is thermodynamically favourable as compared to the (*Z*)‐isomer. The activation barrier of the neutral compound is approximately 1.0 eV. By charging the azothiophene (positively or negatively), the activation barrier for the back‐conversion is lowered drastically to ca. 0.1 eV. From these calculations, we predict that both an oxidative and a reductive reaction channel should be feasible to trigger the back‐conversion from (*Z*) to (*E*)‐isomer electrochemically. This is similar to what was previously observed for ABs.[^17^, ^31^]

**Figure 2:**
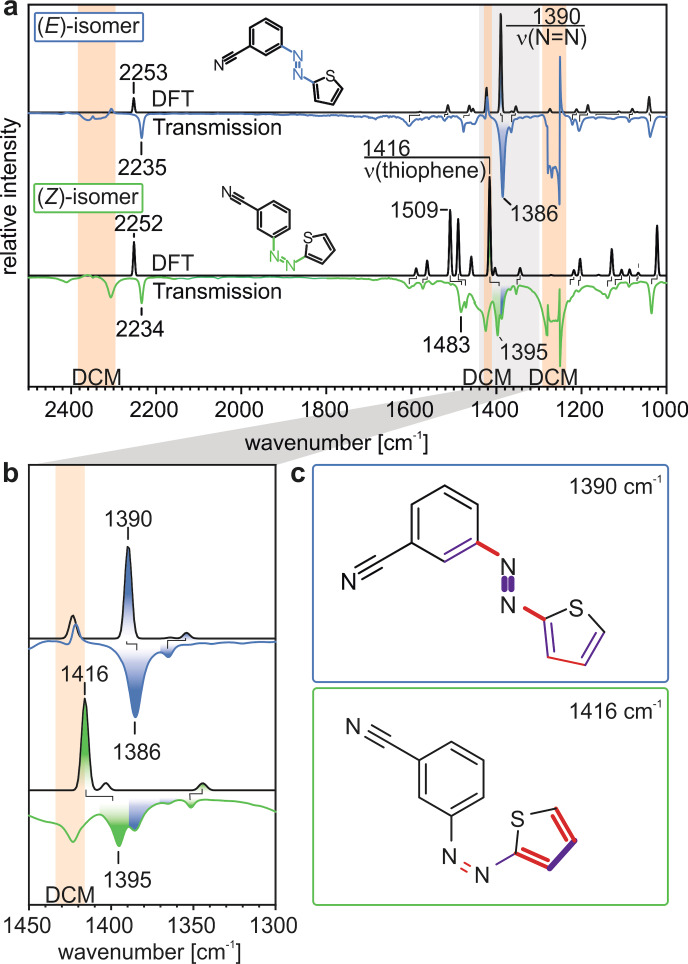
Assignment of the IR bands of (*E*)‐ and (*Z*)‐3‐cyanophenylazothiophene to the corresponding vibrational modes. a,b) Transmission spectra of (*E*) (blue) and (*Z*)‐3‐cyanophenylazothiophene (green) and the corresponding calculated spectra (black). c) Visualization of the vibrational modes of the bands at 1390 cm^−1^ and 1395 cm^−1^. Note that a small fraction of the (*E*)‐isomer was present in the solution of the (*Z*)‐isomer.

To identify the different species in the in situ experiment, we calculated in the next step the IR spectra of the (*E*) and the (*Z*)‐isomer by DFT and assigned the experimental IR bands (recorded in transmission geometry in DCM) to the different vibrational modes (see Figure 2). We summarize the band assignment in Tables 1 and 2 for the (*E*) and (*Z*)‐3‐cyanophenylazothiophene, respectively. Note that in the spectrum of the (*Z*)‐isomer there are also features present of the remaining (*E*)‐isomer (marked in blue). Additional features at 1265 cm^−1^, 1420 cm^−1^ and 2350 cm^−1^ are related to the solvent DCM.^[32]^

**Table 1:** Experimental and calculated IR bands of (*E*)‐3‐cyanophenylazothiophene and corresponding vibrational modes shown in Figure 2.

ν_experimental_ [cm^−1^]	ν_calculated_ [cm^−1^]	Vibrational modes^[a]^
2235	2253	ν (CN)
1609	1580	ν (CC)_phenyl_
1523	1515	ν (CC)_thiophene_
1477	1464	δ (CH)_phenyl_, δ (CC)_CN_
1386	1390	ν (NN)
1364	1354	ν (CC)_phenyl_, δ(CH)_phenyl_, δ(CC)_CN_
1223	1212	δ (CH)_thiophene_, δ (CC)_CN_
1205	1185	δ (CH)_thiophene_, δ (NNC), δ (CC)_CN_
1086	1081	δ (CCN), δ (CH)_phenyl_
1038	1041	δ (CH)_thiophene_, δ (CC)_CN_

[a]. Based on the DFT calculations.

**Table 2:** Experimental and calculated IR bands of (*Z*)‐3‐cyanophenylazothiophene and corresponding vibrational modes shown in Figure 2.

ν_experimental_ [cm^−1^]	ν_calculated_ [cm^−1^]	Vibrational modes^[a]^
2234	2252	ν (CN)
1608	1589	ν (CC)_phenyl_
1574	1562	ν (CC)_phenyl_
1483	1509	ν (NN‐CC)_azo‐thiophene_
1473	1490	ν (NN‐CC)_azo‐thiophene_
1395	1416	ν (CC)_thiophene_
1352	1345	ν (CH)_thiophene_
1229	1218	δ (CH)_thiophene_
1207	1204	δ (CH)_thiophene_
1138	1129	δ (CH)_thiophene_
1121	1106	δ (CH), δ (CCN)
1087	1087	δ (CCN), δ (CH)_phenyl_
1066	1066	δ (CH)
1036	1022	δ (CH)_thiophene_, δ (CC)_CN_

[a]. Based on the DFT calculations.

In specific, we highlight the bands at 1386 cm^−1^ (*ν*(N=N)) and 1395 cm^−1^ (*ν*(thiophene)), which are characteristic for the (*E*) and (*Z*)‐isomer, respectively (see Figure 2b and c). In the following, we use these bands as spectroscopic markers, which enable us to identify the isomers and quantify their concentration (see Supporting Information for details).

**Figure 3:**
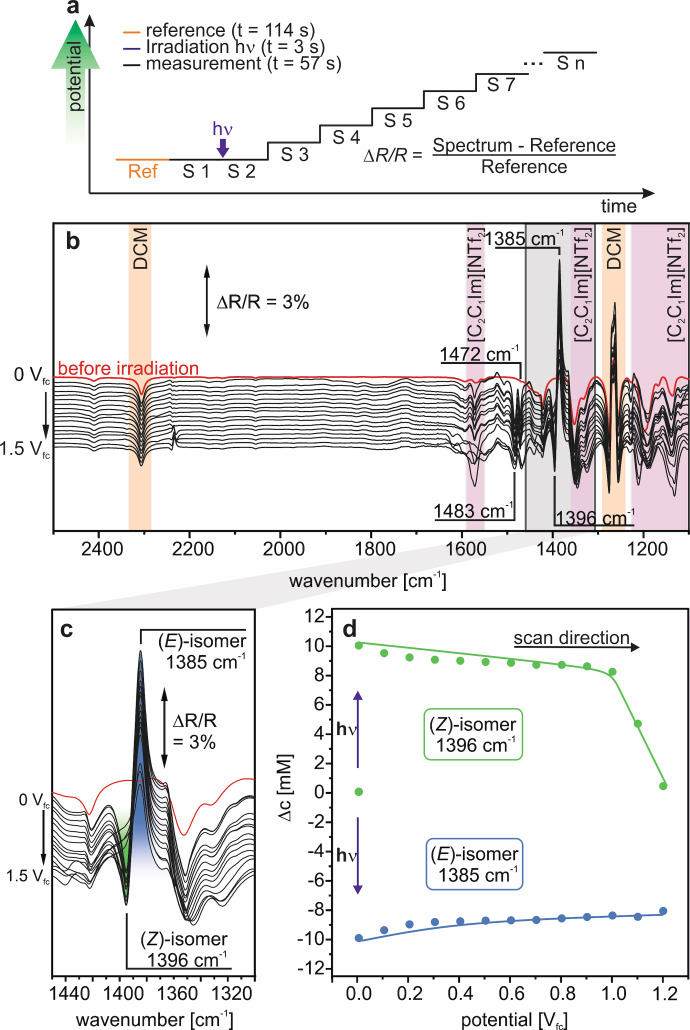
Oxidative reaction channel. a) Experimental procedure. b) PEC‐IRRA spectra. c) Region of the spectroscopic marker. d) Corresponding changes in concentrations for the (*E*)‐ and (*Z*)‐isomer during the experiment as derived from the band intensities of the spectroscopic marker. The reference spectrum was recorded at 0 V_fc_.

In the next step, we studied the photochemical conversion and, subsequently, the oxidatively triggered back‐conversion. To this aim, we first recorded a reference spectrum (2 min acquisition time) at 0.0 V_fc_ and, subsequently, spectra before and after irradiation at different electrode potentials (3 s, 1 min acquisition time). We increase the potential in 0.1 V steps up to 1.5 V_fc_ and measured the corresponding spectra at each potential step (see Figure 3a). The potential windows investigated for both, the oxidative and the later discussed reductive pathway were chosen according to the stability window of the solvent DCM.^[33]^

In Figure 3b we show the resulting data. After irradiation, we observe negative bands at 1574 cm^−1^ (*ν*(CC)_ring_), 1483 cm^−1^ (*ν*(CC)_ring_), and 1396 cm^−1^ (*ν*(thiophene)) which we assign to the formation of the (*E*)‐isomer and a positive band at 1385 cm^−1^ (*ν*(NN)), which we attribute to the consumption of the (*Z*)‐isomer. Note that positive (pointing upwards) and negative (pointing downwards) bands indicate consumed and formed species, respectively. Other bands at 1574 cm^−1^ (*ν*(C=N)_ring_, *ν*(C=C)_ring_), 1350 cm^−1^ (*ν*_as_(SO_2_)), 1200 cm^−1^ (*ν*([NTf_2_]^−^)), and 1135 cm^−1^ (*ν*_s_(SO_2_), *ν*_as_(CF_3_)) we attribute to the supporting electrolyte [C_2_C_1_Im][NTf_2_]^[34]^ (for details of the ATR‐IR spectrum see Supporting Information) and bands at 2309 cm^−1^ and 1260 cm^−1^ to the solvent DCM.^[32]^ Time‐resolved spectra (see Supporting Information Figure S2) show that under our experimental conditions we obtain a quantitative photo‐conversion of the photoswitch in the thin‐layer within a few tenths of a second.

Between 0.1 V_fc_ and 1.0 V_fc,_ the intensities of the peaks attributed to the (*E*) and (*Z*)‐isomer (see above) remain nearly stable and decrease rapidly at potentials above 1.0 V_fc_. In the frequency region of our spectroscopic marker (see Figure 3c), we observe that the band attributed to the (*Z*)‐isomer at 1396 cm^−1^ totally disappears between 1.3 V_fc_ to 1.5 V_fc_, while the positive band at 1385 cm^−1^ (attributed to the consumed (*E*)‐isomer) remains. Also, we observe the appearance of additional peaks at 1441 cm^−1^, 1466 cm^−1^, and 1550 cm^−1^, which might result from decomposition products. However, we are not able to assign the bands to specific species. For a detailed analysis of the data, we performed a quantitative analysis based on the band intensities and plotted the concentrations against the applied potential (see Figure 3d).

When irradiating the solution at 0.0 V_fc_, the (*E*)‐isomer is photo‐converted to the (*Z*)‐isomer quantitatively. By increasing stepwise the potential from 0.0 V_fc_ to 1.0 V_fc,_ a small fraction (18 %) of the formed (*Z*)‐isomer converts back to the (*E*)‐isomer. We assign this effect to a thermally triggered isomerization within the thin‐layer. At 1.1 V_fc_, we observe the onset potential for the oxidation of the (*Z*)‐isomer leading to decomposition. However, no electrochemically triggered back‐conversion is detected (see selected spectra in Figure SI6). We conclude that the onset potential of the oxidative decomposition is below the onset of the electrochemically triggered back‐conversion.

Previously, Goulet‐Hanssens et al. showed for azobenzenes that the potential differences between reversible oxidation (1‐electron transfer) triggering the back‐conversion and an irreversible oxidation (2‐electron transfer leading to decomposing the photoswitch) are close to each other.^[27]^ This limits the selectivity of the oxidative channel for the electrochemically triggered energy release from azobenzenes. Our results indicate that similar limitations apply for azothiophenes.

**Figure 4:**
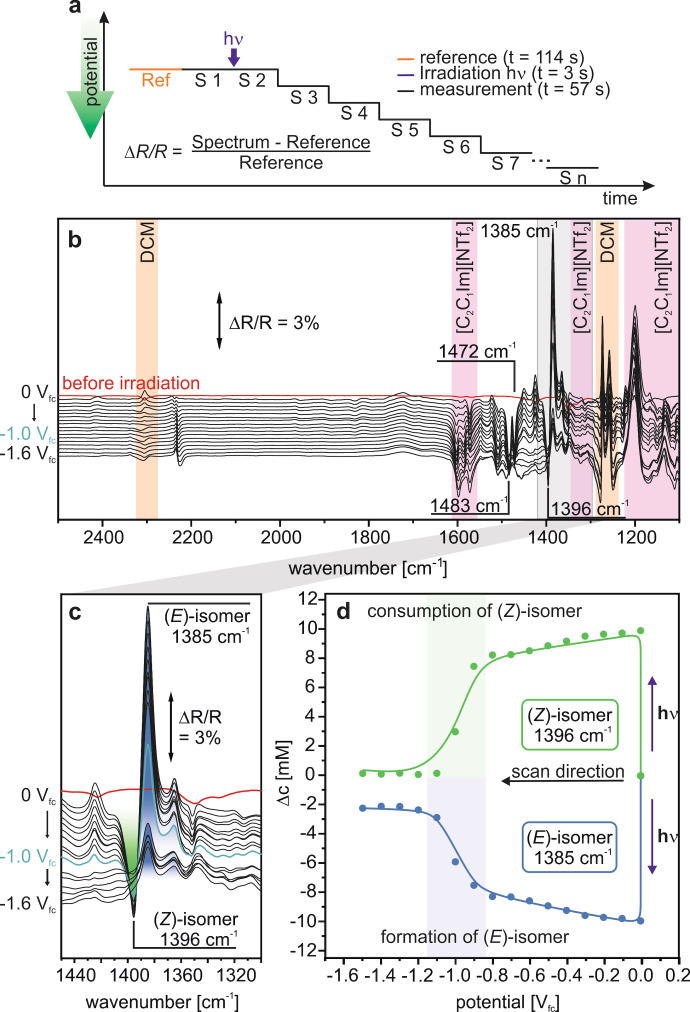
Reductive reaction channel. a) Experimental procedure. b) PEC‐IRRA spectra. c) Region of the spectroscopic marker. d) Corresponding changes in concentrations for the (*E*)‐ and (*Z*)‐isomer during the experiment as derived from the band intensities of the spectroscopic marker. The reference spectrum was recorded at 0 V_fc_.

Furthermore, we investigated the reductive reaction pathway. Initially, we irradiated the thin‐layer at 0.0 V_fc_ and, subsequently, decreased stepwise the potential from 0 V_fc_ to −1.5 V_fc_ in 0.1 V steps (see Figure 4a). After each step, we measured EC‐IRRA spectra, as shown in Figure 4b. After irradiation, positive bands appear at 1385 cm^−1^ and negative bands at 1574 cm^−1^, 1483 cm^−1^, and 1396 cm^−1^, which we assigned to the (*E*) and (*Z*)‐isomer (see above), respectively. The positive and negative bands strongly decrease or vanish at potentials≤−0.9 V_fc_, as illustrated for the spectroscopic marker bands at 1385 cm^−1^ and 1396 cm^−1^ (Figure 4c). The spectra indicate that both the photochemical conversion and the electrochemically triggered back‐conversion are efficient. A quantitative analysis of the spectra is shown in Figure 4d. In contrast to the oxidatively triggered back‐conversion, consumption of the (*Z*)‐isomer and formation of the (*E*)‐isomer occur in parallel with an onset potential of −0.9 V_fc_. This observation demonstrates that the reductive energy release channel is not affected by a competing reduction channel that leads to decomposition. The back‐conversion can be triggered electrochemically between −0.9 V_fc_ and −1.5 V_fc_. In the presented potential‐step experiment, we reach a selectivity (*S(E)*=−Δ*c(E)*/Δ*c(Z)*) of 0.8 for the reductively triggered back‐conversion. Note that we investigated the stability of the MOST systems under electrochemical conditions in more detail in a repeated cycling experiment, which we describe later in this work.

**Figure 5:**
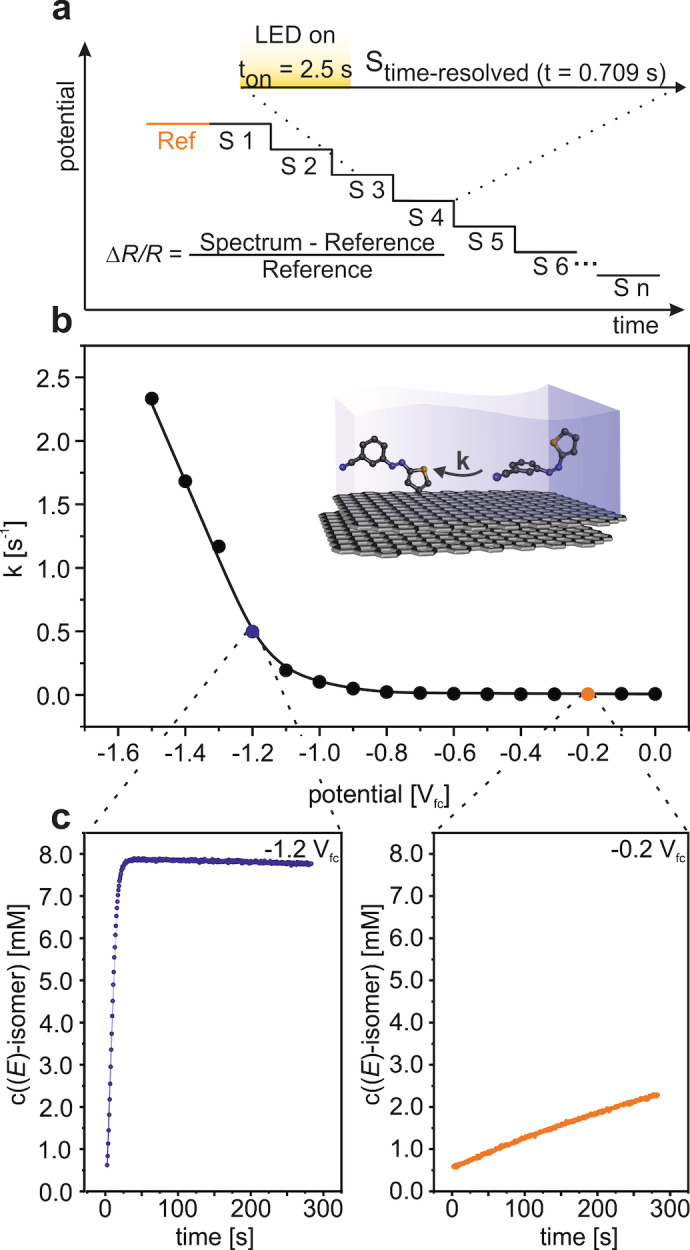
Potential dependent kinetics of the reductive isomerization reaction. a) Experimental procedure. b) Reaction constant depending on the potential. c) Time dependent development of concentration for −0.2 and −1.2 V_fc_.

In the next step, we addressed the reaction kinetics of the reductive reaction channel. In the experiment, we recorded time resolved spectra at potentials ranging from 0 V_fc_ to −1.5 V_fc_ (see Figure 5a). We recorded a reference and one additional spectrum at 0 V_fc_. Subsequently, we irradiated the thin‐layer for 2.5 s. After switching the LED off, we recorded spectra (in total 4.7 min) with a time resolution of 709 ms/spectrum at the different potentials. From the development of the concentration, we determined the rate constant as a function of the potential (shown in Figure 5b). As an example, we plotted the development of the concentrations for two potentials in Figure 5c (see the corresponding spectra in Figure S7 in the Supporting Information). The rate constant remains low (<0.01 s^−1^) from 0 V_fc_ to −0.8 V_fc_, indicating that there is slow thermal back‐conversion only. Below −0.9 V_fc_, the rate constant increases drastically with decreasing potential and reaches a value of 2.33 s^−1^ at −1.5 V_fc_. We attribute this behaviour to the onset of reductively triggered back‐conversion. The results imply that it is possible to control the rate of the energy release via the potential applied.

**Figure 6:**
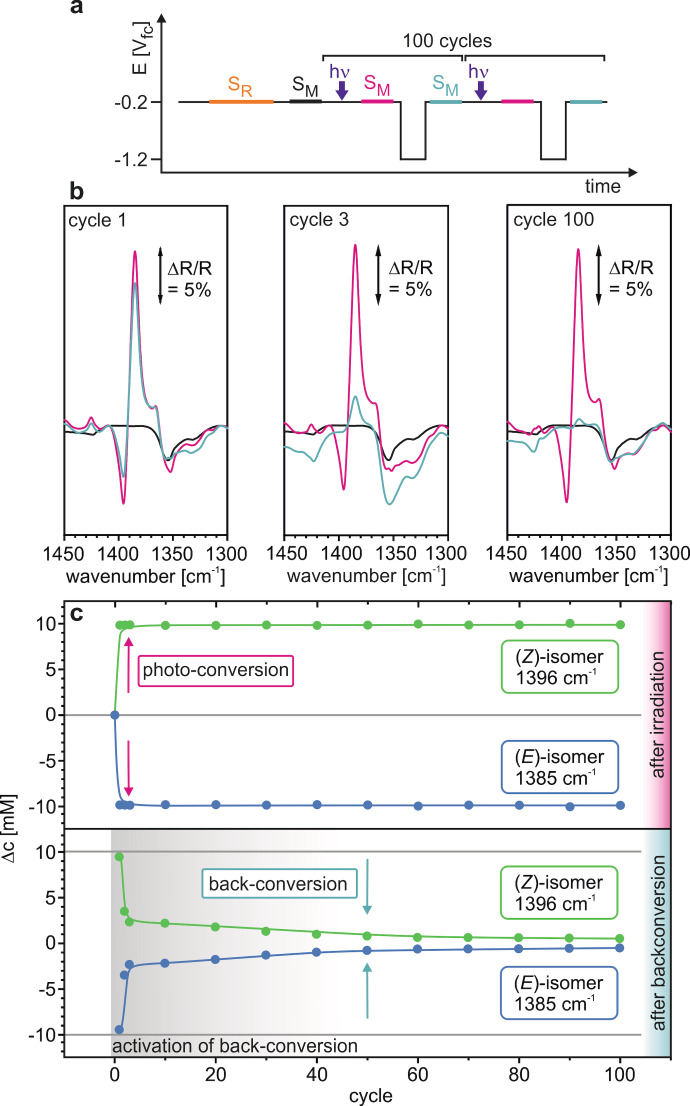
Stability test of the electrochemically triggered (*E*)/(*Z*)‐3‐cyanophenylazothiophene energy storage system. a) Experimental procedure covering 100 conversion cycles. b) Selected PEC‐IRRA spectra (cycle 1, 3, and 100) in the region of the spectroscopic marker before (black) and after irradiation (red) and after electrochemically triggered back‐conversion (blue). c) Cycle dependent development of the concentration of the (*E*)‐ and (*Z*)‐isomer with respect to the starting concentration. The reference spectrum was recorded at −0.2 V_fc_.

Finally, we investigated the reversibility of the storage process. We performed 100 storage and release cycles, each comprising of irradiation at −0.2 V_fc_ (photochemical conversion, 3 s) and energy release after a potential jump to −1.2 V_fc_ (electrocatalytically triggered back‐conversion, 60 s) as illustrated in Figure 6a. Between each step, we measured spectra (S_M_) at −0.2 V_fc_. Note that mass exchange between the thin‐layer and the bulk solution is very slow and occurs on the timescale of several hours only.[^12^, ^35^] All spectra refer to the reference spectrum (S_R_) measured before the 100 cycles at −0.2 V_fc_. In Figure 6b, we show the resulting spectra in the region of the spectroscopic marker after cycle 1, cycle 3, and cycle 100. After irradiation (red spectra), we observe the formation of the positive band at 1385 cm^−1^ and the negative band 1395 cm^−1^, characteristic for the (*E*)→(*Z*) isomerisation. The intensity of these spectra remains identical over 100 cycles. The development of the bands after the potential jump (blue spectra) is more complex. While the bands only slightly decrease in the first cycle, they vanish nearly completely in the following cycles. This observation indicates that the electrochemically triggered back‐conversion becomes more efficient over time. Interestingly, the effect is getting more and more pronounced with increasing cycle number within the 100 cycles tested. We conclude that after a first activation phase the electrochemical back‐conversion occurs with very high efficiency and selectivity. We attribute the activation phase to poisoning impurities which may originate from the environment, from the synthesis procedure, or from the equipment used. They are removed reductively during the potential cycling. Figure 6c shows the results of the corresponding quantitative analysis. We display the change of concentrations (Δ*c*) of the (*E*) and (*Z*)‐isomer with respect to the starting concentration after irradiation (top) and after the potential jump (bottom). After irradiation, the photoswitch is converted quantitatively and selectively over the duration of all 100 cycles. After the initial potential jump, only 5 % of the (*Z*)‐isomer is converted back to the (*E*)‐isomer. Thereafter, the activity of the system increases rapidly so that after three cycles approximately 80 % of the (*Z*)‐isomer is back‐converted. After ca. 50 cycles, the activation process is practically completed and we reach a back‐conversion level of 94 %. Most importantly, the concentration of the (*Z*)‐isomer after irradiation does not decrease within the 100 cycles, which demonstrates that the photoswitch does not decompose under the experimental conditions applied. These results differ somewhat from the potential‐step experiment described in Figure 4, where we observed some minor decomposition. We attribute this difference to the different potential switching procedure, which may strongly influence the electrochemical processes at the electrode.^[36]^ When we decrease the potential stepwise, we expose also the MOST system to intermediate potentials (between −0.8 and −1.1 V_fc_). Under these conditions, the back‐conversion is slower and we might speculate that reactive intermediates formed under these potentials might promote undesired side reactions. This observation shows that for a reversible operation of the storage cycle, not only the MOST storage couple and the electrochemical environment (electrode and electrolyte), but also the electrochemical operation procedure is of importance.

In general, our results show that it is possible to trigger the energy release electrochemically from a MOST system in an efficient way. The approach has several advantages, compared to other energy release strategies. As already small concentrations of intermediates initiate a chain reaction,[^2^, ^12^, ^17^, ^26^] the energy input to trigger the energy release is very low. This results in a higher overall efficiency of the system. As the reaction rate of the back‐conversion is highly dependent on the applied potential, the approach offers a high level of control over the rate of energy release. The present work and previous studies[^11^, ^12^] demonstrate that the approach allows us to reach very high reversibility for different classes of MOST systems. Such high reversibility is essential for the implementation into devices. Finally, the electrochemical pathway may enable a simplified reactor design as compared to catalytic flow reactors and the use of highly concentrated solutions with higher viscosity and higher energy storage capacity.

## Conclusion

We investigated the photochemical conversion and the electrochemically triggered back‐conversion of the isomer couple (*E*)/(*Z*)‐3‐cyanophenyazothiophene for energy storage in MOST systems. In our study, we used photoelectrochemical infrared reflection absorption spectroscopy (PEC‐IRRAS) combined with density functional theory (DFT). We assigned the IR bands of the isomer couple to the corresponding vibrational modes and characterized the oxidative and reductive reaction channels of the electrochemically triggered back‐conversion. In addition, we studied the kinetics of the back‐conversion reaction and tested the stability of the MOST system over 100 storage cycles. From our observations, we conclude:

1. *Reaction channels*: Theory predicts two reaction channels for triggering the (*Z*)→(*E*) isomerization electrochemically, an oxidative (hole‐catalyzed) and a reductive (electron‐catalyzed) channel. In both reaction channels, the activation barrier is predicted to decrease from 1.0 eV for the uncharged molecule to 0.1 eV for the (positively or negatively) charged molecule. While the reductive reaction channel is accessible in the experiment, the photoswitch decomposes during the oxidative reaction channel. We conclude that the onset potential for decomposition (1.1 V_fc_) is below the onset potential for back‐conversion.
2. *Reductive reaction channel*: The onset potential for the reductive reaction channel is −0.9 V_fc_. In the reductive reaction channel, the rate of the back‐conversion reaction can be controlled by the applied potential within (at least) two orders of magnitude between the potentials of 0 V_fc_ and −1.5 V_fc_.
3. *Stability of the MOST system*: In tests over 100 storage cycles we showed that photochemical conversion occurs quantitatively. The electrocatalytic back‐conversion via the reductive reaction channel requires an activation phase, which most likely result from poisoning impurities in the system. After three storage cycles, we reach a conversion of 80 %. After repeated cycling (≈50 cycles), electrochemically triggered back‐conversion of up to 94 % of the (*Z*)‐isomer is possible. Most importantly, we do not observe any decomposition of the photoswitch after 100 conversion cycles.

Our results demonstrate the advantage to trigger the energy release of thiophene‐based MOST systems via the electrochemical pathway. First, the approach enables us to control the reaction kinetics. Secondly, the approach provides excellent cyclability. We expect that electrochemically triggered energy release is also possible for other heteroarylazobenzene‐based MOST systems.

## Experimental Section

### Cleaning

All glass and Teflon ware, as well as all noble metal wires were stored in a solution of NOCHROMIX® (Sigma Aldrich) in concentrated sulfuric acid (Merck, Emsure, 98 %) overnight. Afterwards, we rinsed the equipment 5 times with ultra‐pure water (MilliQ Synergy UV, 18.2 MΩ cm at 25 °C, TOC <5 ppb) and boiled it 3 times in ultra‐pure water for 30 min. Finally, we dried the equipment overnight in an evacuated desiccator.

### PEC‐IRRAS

We performed all PEC‐IRRAS experiments with a vacuum‐based Fourier transform infrared (FTIR) spectrometer (Bruker, Vertex 80v) with evacuated optics, a liquid‐nitrogen‐cooled mercury cadmium telluride (MCT) detector, a commercial optics for electrochemical experiments (Bruker), and a homebuilt electrochemical cell. An ultra‐violet (UV) light emitting diode (LED; Seoul Viosys, CUN6AF4 A, 365 nm, 2350 mW) served as UV‐light source, which was placed below the IR and UV transparent CaF_2_ (Korth, *d*=25 mm) window. We used a KRS‐5 filter to shield the IR detector from UV light. A detailed description of the used setup we provide in our previous work.^[13]^ All in situ measurements were performed in reflection mode in thin‐layer configuration. IR spectra were recorded with a resolution of 2 cm^−1^. We measured reference spectra with 256 scans and other spectra with 128 scans per spectrum (acquisition time 57 s). For time‐resolved measurements, we increased the scanner velocity from 40 kHz to 240 kHz. Using a resolution of 8 cm^−1^, the acquisition time was lowered to 21 ms per scan. We varied the number of scans resulting in different acquisition times of the spectra (see figure captions for details). We used non‐polarized light for all measurements. We measured transmission spectra of the used materials with a commercial liquid transition cell (PIKE Technologies).

All PEC‐IRRAS experiments were performed using highly oriented pyrolytic graphite (HOPG) (MikroMasch, ZYA, 0.4° mosaic spread) as working electrode. Prior to each measurement, we prepared the surface by cleavage using the “scotch tape methode”.[^37^, ^38^] For all experiments, we used a solution of 10 mm (*E*)‐3‐cyanophenylazothiophene in DCM (Sigma‐Aldrich, ≥99.8 %) with 0.1 m 1‐ethyl‐3‐methylimidazoliumbis(trifluoromethylsulfonyl)imide ([C_2_C_1_Im][NTf_2_]) as supporting electrolyte. The potential was controlled by a commercial potentiostat (Gamry, Reference 600) with a three‐electrode configuration. We used a graphite rod as counter electrode and a platinum wire as (pseudo)‐reference electrode. We calibrated the reference electrode in an external cell versus the cyclic voltammogram of ferrocene (Alfa Aesar, 99.5 %). In this work, we refer all potentials measured to the redox potential of the ferrocene couple (V_fc_).

### Density functional theory (DFT)

All calculations were performed in the QChem 5.2 software package.^[39]^ All geometries were optimized at the PBE‐D3(BJ)/def2‐TZVP level of theory[^40^, ^41^, ^42^] and verified as minimum (with zero imaginary frequencies) or transition state (with exactly one imaginary frequency) via subsequent frequency analysis which also were used to obtain IR spectra. Transition states (TS) were validated by internal reaction coordinate (IRC) calculations to identify the minima connected by the respective TS. The calculations were performed for gas phase molecules at 0 K. The calculated spectra were compared with transmission IR spectra to validate the theoretical method for the ground‐state calculations.

### Synthesis of (*E*)‐3‐cyanophenylazothiophene

To a solution of *i*PrMgCl⋅LiCl (1.3 mol L^−1^ in THF, 6.1 mL, 8.0 mmol, 2.0 equiv.) in a dry Schlenk‐tube under nitrogen atmosphere, neat 2‐bromothiophene (1.0 g, 6.0 mmol, 1.5 equiv.) was added dropwise at room temperature (RT). After stirring for 45 min, the solution was diluted with dry THF (5 mL) and cooled to −20 °C. Then, ZnBr_2_ in dry THF (1.0 mol L^−1^, 3.2 mL, 3.2 mmol, 0.80 equiv.) was added dropwise. The mixture was warmed to RT and stirred for 15 min. Following, the dithiophenylzinc solution was added dropwise to a solution of 3‐cyanobenzenediazonium tetrafluoroborate (0.87 mg, 4.0 mmol, 1.0 equiv.) in dry THF/NMP, (1 : 1, 28 mL) at −40 °C overnight. Afterwards, the suspension was allowed to warm to −20 °C and was stirred at this temperature for additional 6 h. The reaction was quenched by the addition of sat. aq. NH_4_Cl (10 mL) and water (40 mL). After dilution with Et_2_O (40 mL) and phase separation, the aqueous phase was extracted with Et_2_O (2x 40 mL) and the combined organic phases were washed with water and brine (50 mL each). After drying over MgSO_4_, filtration and evaporation of the solvents, the residue was adsorbed on silica gel (4.0 g) and was purified by flash column chromatography (70 g SiO_2_, Cyc/EtOAc/DCM; 15 : 1 : 1) to yield an orange solid (0.29 g, 34 %). For further details on the synthesis we refer to the literature (Procedure A).^[19]^

^1^H NMR (400 MHz, CDCl_3_) *δ* 8.14 (t, *J=*1.8 Hz, 1 H), 8.08 (ddd, *J=*8.0, 2.0, 1.2 Hz, 1 H), 7.88 (dd, *J=*3.9, 1.3 Hz, 1 H), 7.70 (dt, *J=*7.7, 1.4 Hz, 1 H), 7.60 (t, *J=*7.8 Hz, 1 H), 7.50 (dd, *J=*5.3, 1.3 Hz, 1 H), 7.20 ppm (dd, *J=*5.4, 3.9 Hz, 1 H) m.p. 126 °C.

### Synthesis of 1‐ethyl‐3‐methylimididazolium bis(trifluoromethanesulfonyl)imide [C_2_C_1_Im][NTf_2_]

The ionic liquid was synthetized by ion metathesis, starting from the commercial ethylsulfate (Aldrich) [C_2_C_1_Im][EtSO_4_]. To this purpose 100 g (0.423 mol) of [C_2_C_1_Im][EtSO_4_] were dissolved in (100 mL) acetonitrile (HPLC grade) and precipitated by adding diethyl ether (500 mL) (analytical grade). The oily product was decanted and dried at 50 °C by means of a rotary evaporator. The resulting viscous pale yellow oil was dissolved in deionized water (350 mL) and the solution decolorized with active charcoal. After careful filtration, solid Li[NTf_2_] (1.1 equiv. with respect to the starting sulfate) was added to the colorless solution and the mixture was vigorously stirred for 30 min. After this time, methylene chloride (analytical grade) was added (150 mL) and the stirring was continued for further 15 min. Then, the whole mixture was transferred to a separatory funnel and allowed to settle. The lower organic phase was washed several times with small volumes of deionized water, and then transferred to a rotary evaporator and dried at 80 °C/5 mbar for 6 h. The final product was obtained in 86 % yield as colorless liquid. ^1^H NMR (400 MHz, DMSO‐d_6_) *δ* 0.82 (t, *J*=7 Hz, 3H), 4.12 (quart, *J*=7 Hz, 2H), 7.58 (s, 1H), 7.67 (s, 1H), 9.01 (s, 1H). ^13^C‐{^1^H}: *δ* 14.0, 36.4, 49.6, 120.0 (quart, *J*=319 Hz, *C*F_3_), 122.7, 124.0, 136.8. Karl‐Fisher titration revealed a content of 480 ppm of water.

## Conflict of interest

The authors declare no conflict of interest.

1

## Supporting information

As a service to our authors and readers, this journal provides supporting information supplied by the authors. Such materials are peer reviewed and may be re‐organized for online delivery, but are not copy‐edited or typeset. Technical support issues arising from supporting information (other than missing files) should be addressed to the authors.

Supporting Information

## Data Availability

The data that support the findings of this study are available from the corresponding author upon reasonable request.
