# Supplementary material for: Electrochemically Triggered Energy Release from an Azothiophene‐Based Molecular Solar Thermal System
Source: ChemSusChem. 2022 Jul 27;15(18):e202200958. doi: 10.1002/cssc.202200958 (PMC9796447; doi:10.1002/cssc.202200958)
Supplement: Supplementary file 1 — Supporting Information [file CSSC-15-0-s001.pdf]

ChemSusChem

Supporting Information

Electrochemically Triggered Energy Release from an Azothiophene-Based Molecular Solar Thermal System

Evanie Franz, Anne Kunz, Nils Oberhof, Andreas H. Heindl, Manon Bertram, Lukas Fusek, Nicola Taccardi, Peter Wasserscheid, Andreas Dreuw, Hermann A. Wegner, Olaf Brummel,* and Jörg Libuda© 2022 The Authors. ChemSusChem published by Wiley-VCH GmbH. This is an open access article under the terms of the Creative Commons Attribution License, which permits use, distribution and reproduction in any medium, provided the original work is properly cited.

1. **Energies of the charged and uncharged isomers determined by DFT**

Table S1: Calculated energies of the neutral, positively and negatively charged (*Z*) and (*E*)-isomer and the corresponding transition states of the 3-cyanophenylazothiophene.

Charge	Isomer	Total energy [h]	relative energy with respect to (*Z*)-isomer [h]	relative energy with respect to (*Z*)-isomer [eV]
uncharged	(*E*)-isomer	-985,1454807	-0,019477303	-0,530004681
	(*Z*)-isomer	-985,1260034	0	0
	Transitionstate	-985,0882802	0,037723242	1,026502217
positively charged	(*E*)-isomer	-984,8635809	-0,01231504	-0,335109484
	(*Z*)-isomer	-984,8512659	0	0
	Transitionstate	-984,846526226897	0,004739663	0,128972856
negatively charged	(*E*)-isomer	-985,2095187	-0,022196226	-0,603990393
	(*Z*)-isomer	-985,1873225	0	0
	Transitionstate	-985,182207	0,00511543	0,139198002

2. **Identification of the dominant conformers**

For both, the (*E*) and the (*Z*)-isomer, DFT calculations predict “untwisted” and “twisted” conformers, respectively (see Figure S1a). Note that we defined “twisted” and “untwisted” according to the orientation of the sulfur atom respective to the azo group. The conformers in which the sulfur is in cis and trans position to the azo group are defined as untwisted and twisted, respectively. To identify the conformers, which are dominantly present, we calculated the energies of the corresponding structures. Additionally, we compared the calculated IR spectra of both conformers with the measured IR spectra of 10 mM 3-cyanophenylazothiophene in DCM. Upon geometry optimizations of the (*E*)/(*Z*)-isomers, the “twisted” conformers of both isomers have higher energies by approx. 13 kJ/mol (5 millihartree). This corresponds to a population of the “twisted” form of less than 0.5% (assuming a Boltzmann distribution) while the “untwisted” form is the dominant form (≥99.5%) in thermodynamic equilibrium at room temperature. In Figure S1b we compare the calculated spectra of both conformers with the measured spectra. We marked bands in grey, which can be assigned to both isomers. In red, we highlighted bands specific for one conformer only. These we used to identified the dominant species. For the (*E*)-isomer (Figure S1b, top) we observe in the calculated spectrum of the twisted conformer an intense band at 1333 cm^{-1}, which is attributed to the stretching vibration of the thiophene ring and the nitrogen double bond. This band is nether observable in the calculated spectrum of the “untwisted” conformer nor in the measured spectrum. We conclude, that the (*E*)-isomer is dominantly present as “untwisted”-conformer. For the (*Z*)-isomer, we identified in the measured spectrum a band at 1483 cm^{-1}, which is solely observable in the

calculated spectrum of the “untwisted” conformer at 1509 cm^{-1}. Additionally, we observe an intense band at 1325 cm^{-1} in the calculated spectrum of the “twisted” conformer, which does not appear in the measured spectrum. We conclude that the dominant species for the (Z)-isomer is also the “untwisted”-isomer. The comparison of calculated and experimental spectra confirms qualitatively the predicted distribution by DFT for both isomers.

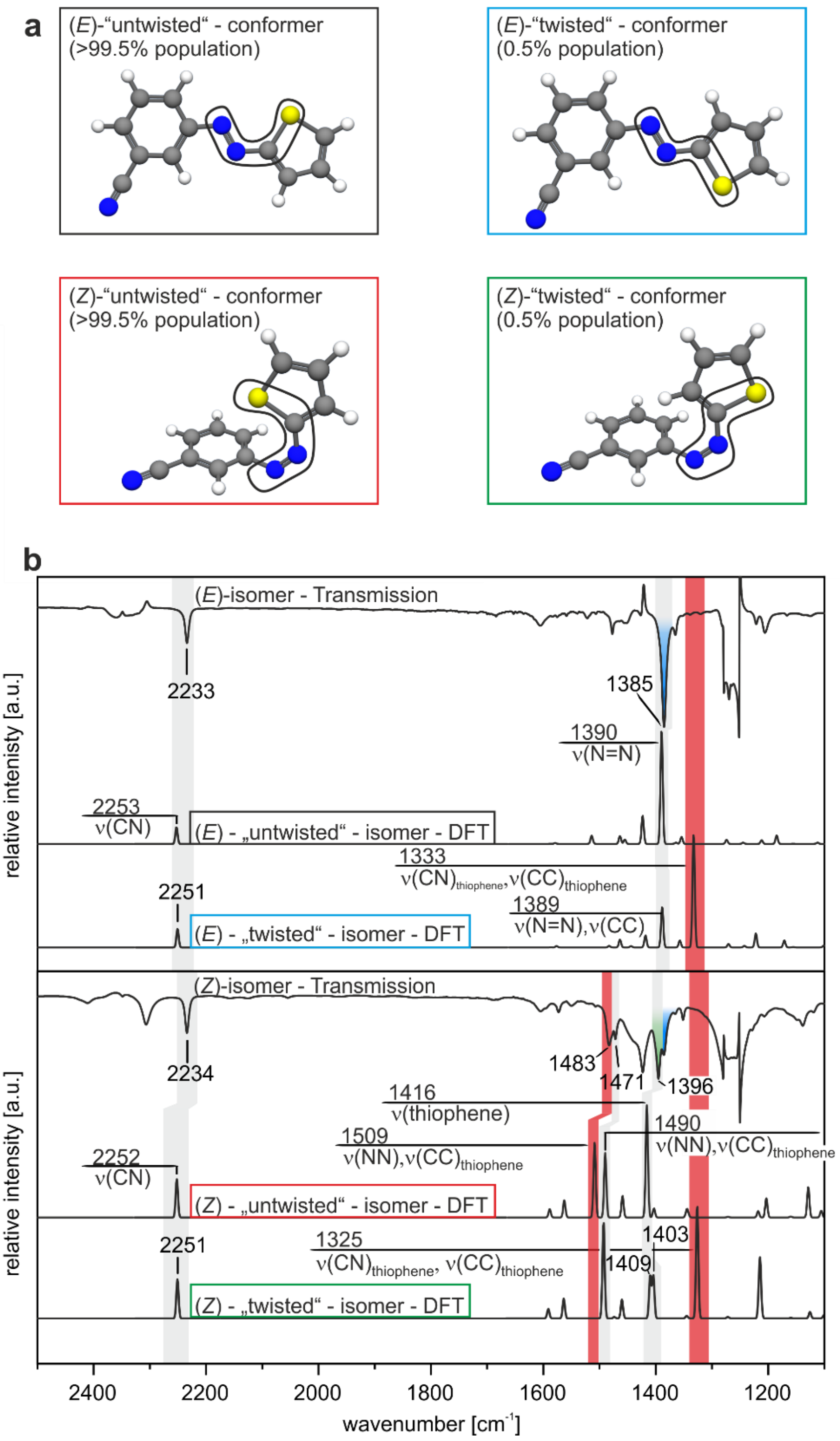

Figure S1: (a) Visualization of possible conformers of 3-cyanophenyazothiophene predicted by DFT; (b) comparison of measured transmission spectra with the calculated spectra of the "untwisted" and "twisted" conformers.

3. Time resolved Photoconversion

We investigated the photochemical conversion by PEC-IRRAS. For this, we recorded a reference spectrum at 0 V_{fc} and subsequently one first spectrum. Afterwards, we irradiated the thin layer and measured spectra with a time resolution of 0.105 s per spectrum (5 scans per spectrum). In Figure S2a we illustrate the measurement procedure. Figure S2b shows the corresponding IR spectra. Note that the spectra are difference spectra; where positive bands (pointing upwards) indicate consumed species and negative bands (pointing downwards) indicate formed species. After irradiation, we observe a positive band at 1385 cm^{-1} and a negative band at 1396 cm^{-1}. Additionally, we observe negative bands at 1472 cm^{-1} and 1483 cm^{-1}. This indicates the consumption of the (*E*)-isomer of the azothiophene-species while the (*Z*)-isomer is formed. Details in the region of the spectroscopic marker are depicted in Figure S2c. In Figure S2d, we plotted the change of concentration with respect to the starting concentration of the (*E*) and (*Z*)-isomer determined from the bands of the spectroscopic marker versus the time. The (*E*)-isomer is quantitatively converted to its energy rich (*Z*)-isomer within 0.2 s. In previous work we determined for the photoisomerization selectivity of >99% in acetonitrile.[1]

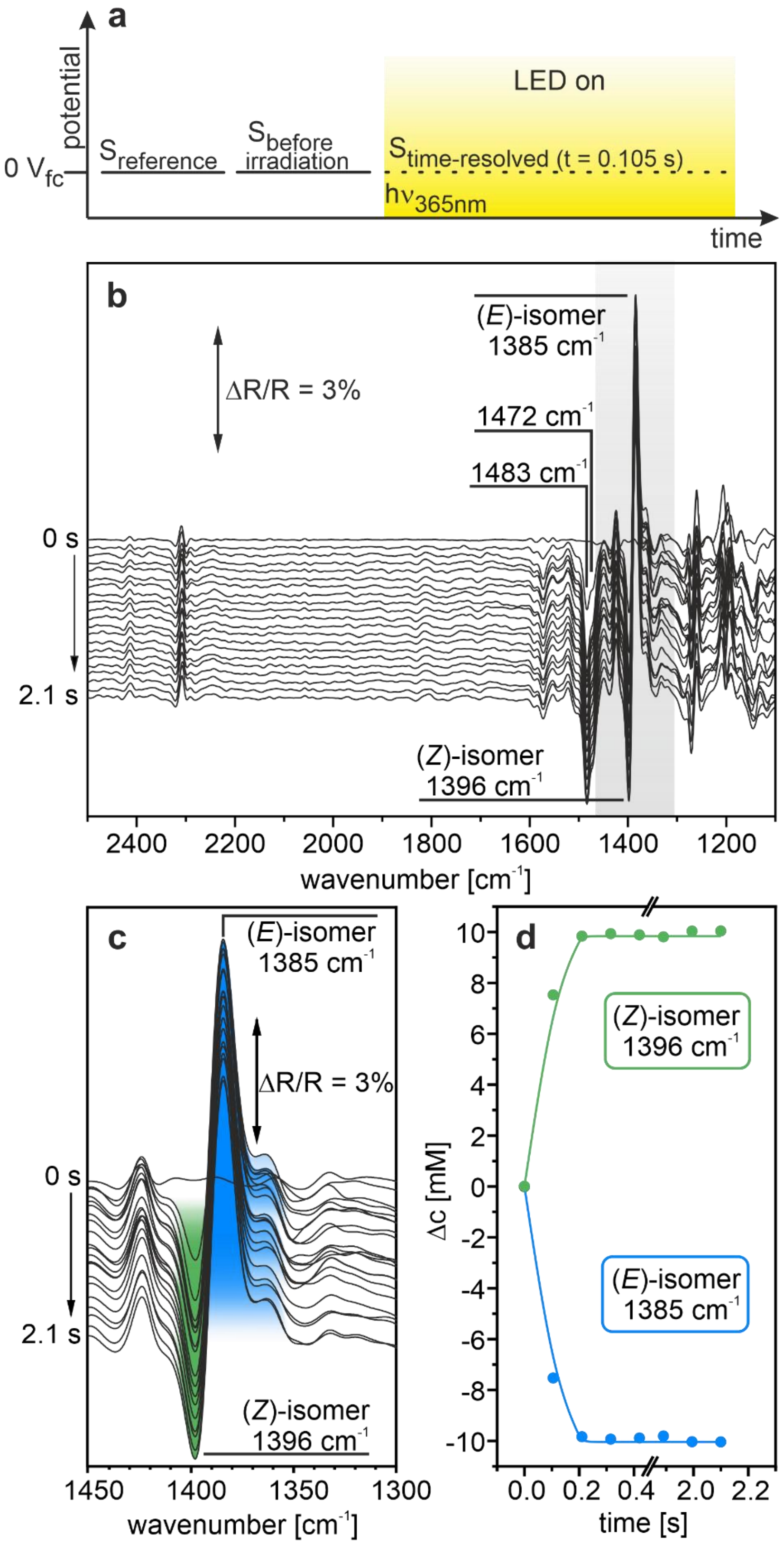

Figure S2: Time-resolved photoconversion; (a) experimental procedure; (b) PEC-IRRA spectra and (c) region of the spectroscopic marker; (d) corresponding changes in concentrations for the (*E*) and (*Z*)-isomer during the experiment as derived from the band intensities of the spectroscopic marker. The spectra were recorded at 0 V_{fc}.

4. ATR-Spectrum of $[C_2C_1Im][NTf_2]$

In Figure S3, we present the full ATR-IR spectrum of the used supporting electrolyte $[C_2C_1Im][NTf_2]$. We assigned the bands according to DFT calculations from previous work.[2]

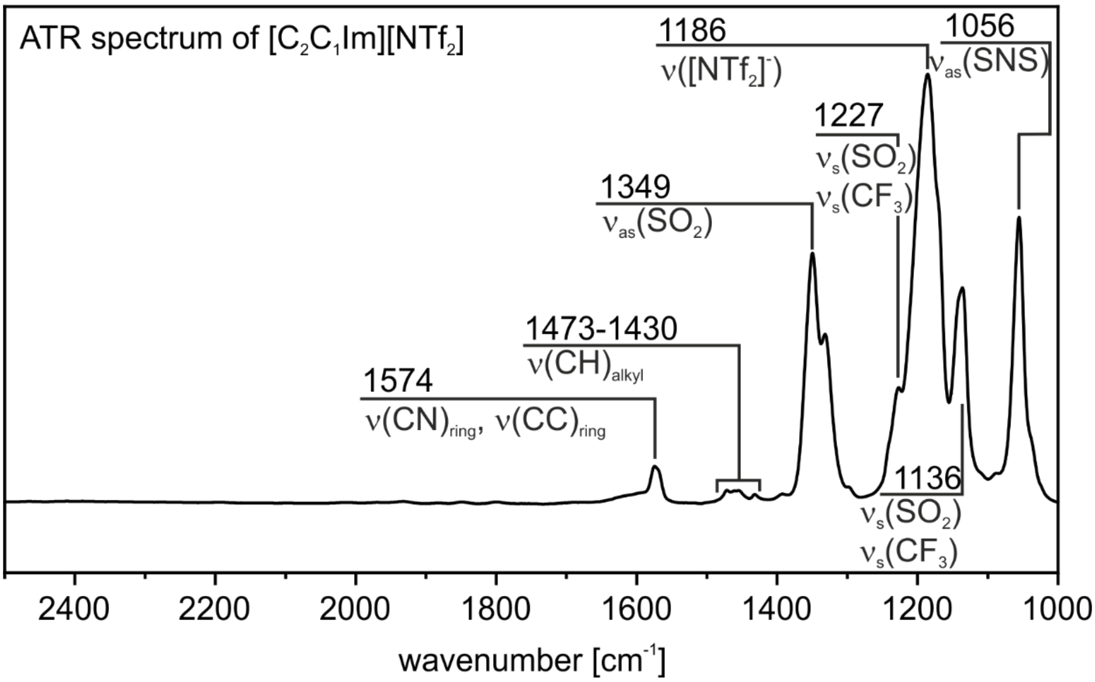

Figure S3: ATR spectrum of the supporting electrolyte $[C_2C_1Im][NTf_2]$.

5. Quantitative Analysis

The concentrations of (*E*)-3-cyanophenylazothiophene and (*Z*)-3-cyanophenylazothiophene were determined according to Beer-Lamberts law

$$\mathrm{A} = \log\frac{I_0}{I} = \varepsilon \cdot d \cdot c$$

Our spectra are recorded as difference spectra, which means that we divide the recorded spectrum by the background spectrum. From this, the absorbance was calculated by

$$\mathrm{A} = -\log\frac{I}{I_0} = \varepsilon \cdot d \cdot c$$

With A = absorbance at 1385 cm^{-1} (for the (*E*)-isomer) and 1396 cm^{-1} (for the (*Z*)-isomer).

With the extinction coefficient ε and the layer thickness d. The concentration of the (*E*)-isomer is known to be 10 mM. We can determine by the absence of a peak at 1385 cm^{-1} after irradiation

in the single channel spectrum, that all compound is converted. By NMR spectroscopy we confirmed that no side products are formed upon irradiation in DCM and all compound is converted to the (*Z*)-isomer. Therefore, the (*Z*)-isomer concentration after irradiation was normalized with respect to the (*E*)-isomer consumption.

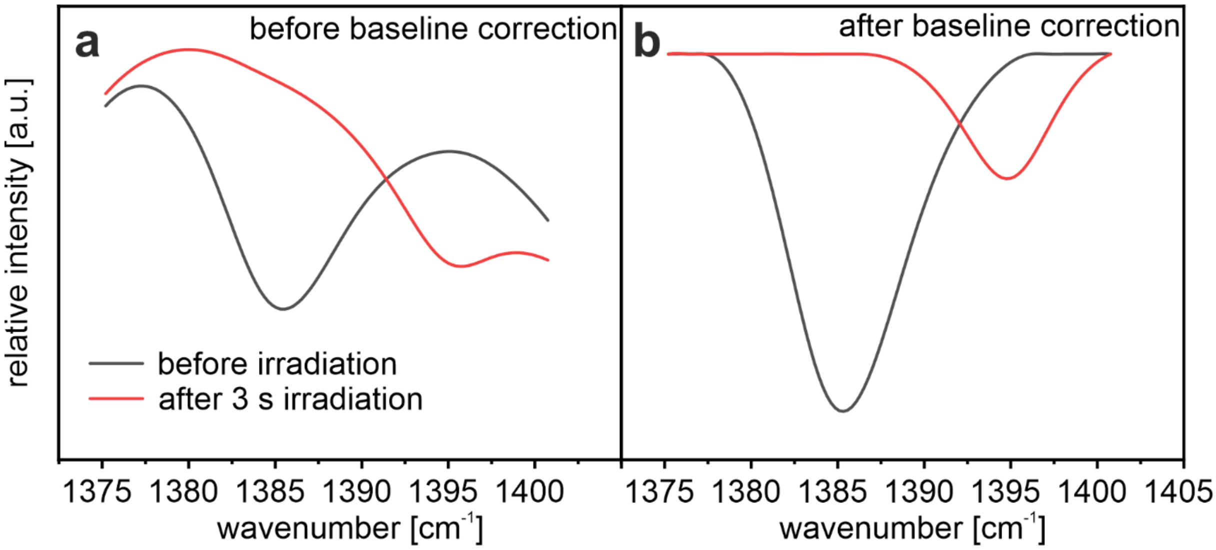

Figure S4: Single channel spectra before and after the photochemical conversion of the (*E*)-isomer to the (*Z*)-isomer at 0 V_{fc} on HOPG; (a) single channel spectrum as measured and (b) after baseline correction.

6. **Stability of 3-cyanophenylazothiophene under operation conditions using different supporting electrolytes**

We tested the stability of azothiophene in DCM under operation conditions using different supporting electrolytes, namely Bu_4NClO_4 (a), Bu_4NBF_4 (b), Bu_4NPF_6 (c), and $[C_2C_1Im][NTf_2]$ (d). In Figure S5, we show the frequency region of the spectroscopic marker. We recorded a reference spectrum (5.4 s acquisition time) at 0 V_{fc} as well as spectra before and after 3 s irradiation. Afterwards, we recorded time-resolved spectra with an acquisition time of 0.709 s. After irradiation, we observe the formation of a negative band at 1398 cm^{-1} and a positive band at 1384 cm^{-1} for all supporting electrolytes, indicating photochemical conversion. For Bu_4NClO_4 and Bu_4NPF_6 (Figure S5a and S5b), the intensity of the negative band decreases over time, while the intensity of the positive band remains constant. This proves that the formed (*Z*)-isomer decomposes over time. We tentatively assign this decomposition to the oxidation of the thiophene and/or the azo moiety. Note that ClO_4^- is known to act as an oxidant and PF_6^- can decompose to oxygenated species, which might act as oxidizers.[3,4] For Bu_4NBF_4, (Figure S5c), however, the formed bands vanish completely after 1 min, demonstrating that the supporting electrolyte efficiently triggers the back-conversion to the (*E*)-isomer. The observed back-

conversion we assign to traces of acids or Lewis acids such as BF_3.[5] If we use the ionic liquid $[C_2C_1Im][NTf_2]$ as supporting electrolyte (Figure S5d) we observe, that the formed bands remain stable over 271 s. This indicates, that the (Z)-isomer is stable under these conditions. As we observe decomposition or back-conversion for the $[Bu_4N]^+$ based supporting electrolytes, we used in our study the ionic liquid $[C_2C_1Im][NTf_2]$ as supporting electrolyte.

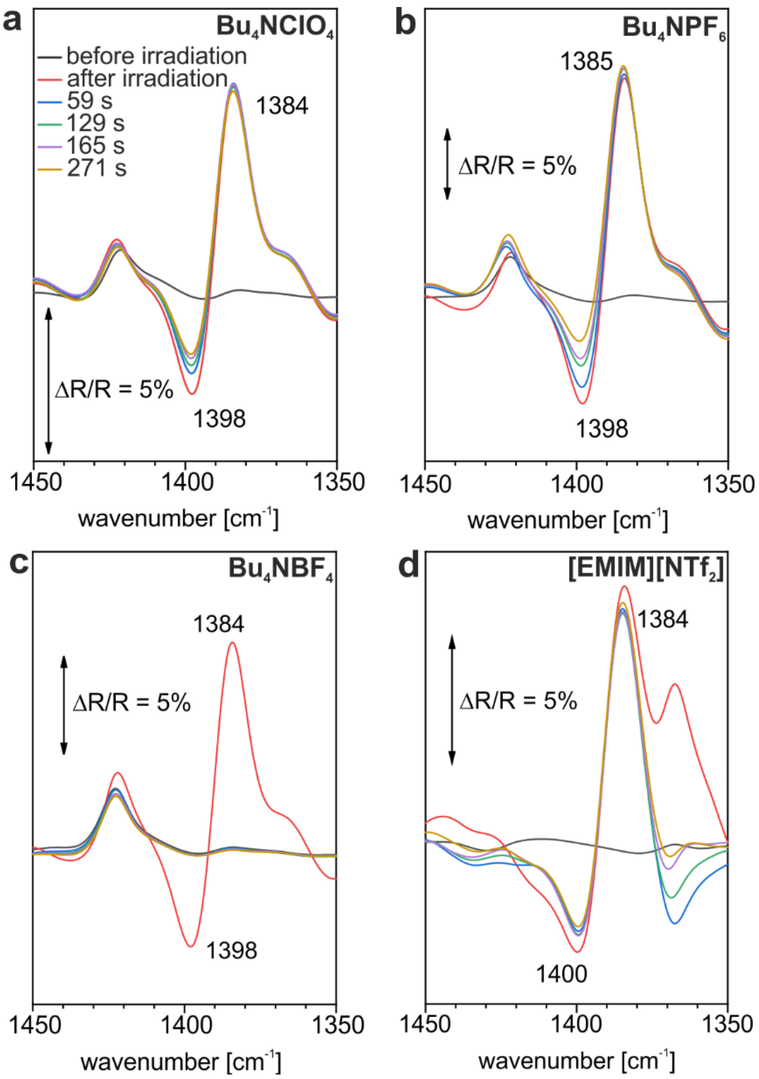

Figure S5: Stability of (*Z*)-3-cyanophenylazothiophene in commonly used supporting electrolytes. Spectroscopic marker region of (*Z*)-3-cyanophenylazothiophene; (a) with 0.1 M Bu_4NClO_4 as supporting electrolyte (b) with 0.1 M Bu_4NBF_4 as supporting electrolyte (c) with 0.1 M Bu_4NPF_6 as supporting electrolyte (d) with 0.1 M $[C_2C_1Im][NTf_2]$ as supporting electrolyte. All spectra were obtained at 0.0 V_{fc}.

7. Selected spectra from the oxidative reaction channel

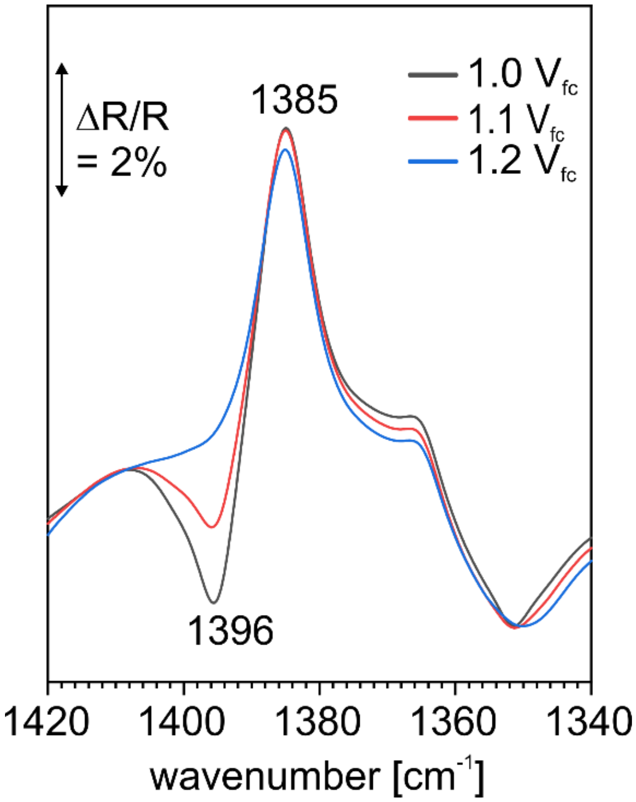

Figure S6: Spectroscopic marker region at 1.0 V_{fc}, 1.1 V_{fc}, 1.2 V_{fc}.

8. Time-resolved spectra at different potentials

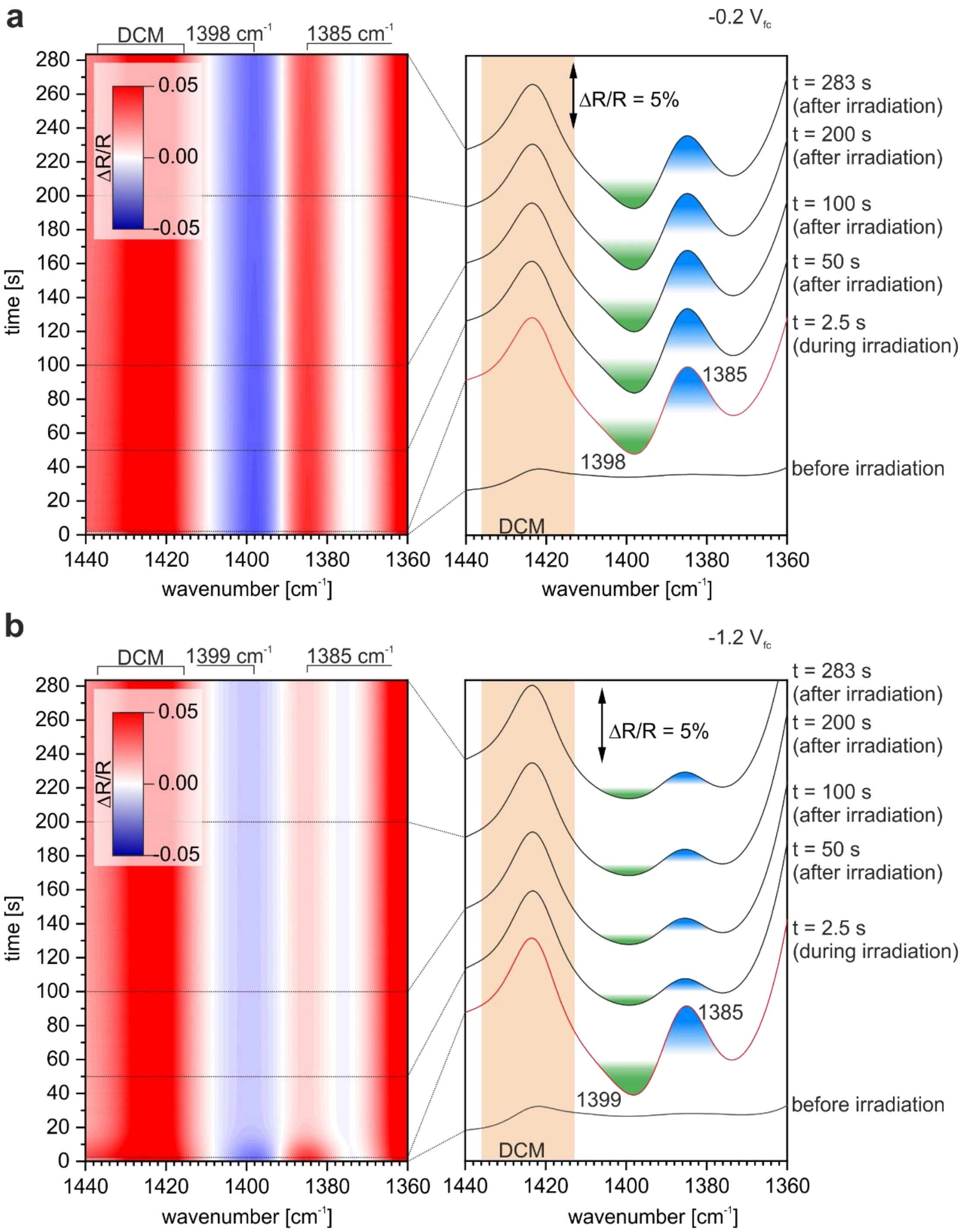

Figure S7: Color plot of the time resolved spectra (left) and selected spectra (right) at -0.2 V_{fc} (a) and -1.2 V_{fc} (b) The reference spectrum was recorded at 0 V_{fc} before irradiation.

References

[1] A. H. Heindl, H. A. Wegner, *Chem. - A Eur. J.* **2020**, *26*, 13730–13737.

[2] C. Hohner, L. Fromm, C. Schuschke, N. Taccardi, T. Xu, P. Wasserscheid, A. Görling, J. Libuda, *Langmuir* **2021**, *37*, 12596–12607.

[3] C. A. Ferreira, S. Aeiyach, M. Delamar, P. C. Lacaze, *Surf. Interface Anal.* **1993**, *20*, 749–754.

[4] A. Goulet-Hanssens, C. Rietze, E. Titov, L. Abdullahu, L. Grubert, P. Saalfrank, S. Hecht, *Chem* **2018**, *4*, 1740–1755.

[5] D. Schulte-Frohlinde, *Über Den Mechanismus Der Katalytischen Cis Zu Trans Umlagerung von Azobenzol*, **1958**.
